# Evaluation of CSF 8-iso-prostaglandin F2α and erythrocyte anisocytosis as prognostic biomarkers for delayed cerebral ischemia after aneurysmal subarachnoid hemorrhage

**DOI:** 10.1038/s41598-024-61956-w

**Published:** 2024-05-17

**Authors:** Karol Wiśniewski, Karol Zaczkowski, Bartosz M. Szmyd, Marta Popęda, Michał Bieńkowski, Bartłomiej Posmyk, Ernest J. Bobeff, Dariusz J. Jaskólski

**Affiliations:** 1 Department of Neurosurgery and Neurooncology, Medical University of Łódź, Kopcińskiego 22, 90-153 Łódź, Poland; 2 Department of Pathomorphology, Medical University of Gdańsk, Gdańsk, Poland; 3 Department of Sleep Medicine and Metabolic Disorders, Medical University of Łódź, Łódź, Poland

**Keywords:** Neuro-vascular interactions, Biomarkers, Neurology

## Abstract

Delayed cerebral ischemia (DCI) is a serious, life-threatening, complication affecting patients who have survived the initial bleeding from a ruptured intracranial aneurysm. Due to the challenging diagnosis, potential DCI prognostic markers should be of value in clinical practice. According to recent reports isoprostanes and red blood cell distribution (RDW) showed to be promising in this respect. We conducted a prospective study of 27 aSAH patients and control group (n = 8). All patients from the study group were treated within the first day of the initial bleeding. We collected data regarding clinical status and results of biochemical, and radiological examinations. We measured cerebrospinal fluid (CSF) concentration of 8-iso-prostaglandin F2α (F2-IsoP) and RDW on days 1, 3, and 5. Both CSF F2-IsoP level and RDW-SD measured on day 1 were significant predictors of DCI. The receiver operating characteristics curve for DCI prediction based on the multivariate model yielded an area under the curve of 0.924 (95% CI 0.824–1.000, *p* < 0.001). In our study, the model based on the combination of RDW and the level of isoprostanes in CSF on the first day after the initial bleeding showed a prognostic value for DCI prediction. Further studies are required to validate this observation.

## Introduction

Aneurysmal subarachnoid haemorrhage (aSAH) is a serious, life-threatening medical condition. Roughly 10% of patients with aSAH die before receiving medical assistance and nearly 40% of them do not survive the first 30 days of hospitalisation^1,2^. Furthermore, one-third of the patients experience severe complications that can result in functional impairment^3^. One of the adverse events present in 20–30% of the SAH patients is delayed cerebral ischemia (DCI)^4,5^. The diagnosis of DCI is based on clinical evaluation, radiological imaging and laboratory tests. DCI is defined as the occurrence of new neurological deficit (e.g., apraxia, aphasia, hemi-inattention, hemiparesis) and/or deterioration in level of consciousness shown as a drop in Glasgow Coma Scale (GCS) of 2 or more points. These symptoms must last for at least 1 h and should not occur following the exclusion of aneurysm from circulation. The diagnosis of DCI is only possible after ruling out other potential causes of the deterioration, such as hypotension, electrolyte imbalance, infection or re-bleeding^6–8^. Although the specific pathomechanism of DCI remains unclear, many authors emphasise that its cause is complex and comprises microcirculatory disturbances, microthrombosis, inflammation, blood-barrier disruption, electrolyte imbalances and cerebral autoregulation disorders.

According to one of the most likely theories, metabolic changes associated with extravasated blood led to an inflammation in the arterial walls and oxidative stress (OS)^9–11^. OS is a condition marked by increased levels of toxic oxygen species (TOS), reactive oxygen species (ROS) and endogenous antioxidants. The elevated concentration of ROS, leads to predominance of oxidation reaction and finally, to disruption of redox signalling (RS)^12^. RS regulates “redox switches” which are diverse mechanisms which influence endothelium and vasoconstriction^13–18^. As a result, there are local perfusion disruptions which may be responsible for the clinical picture of DCI. Since ROS levels have been associated with F2-isoprostanes (F2-IsoPs), both have been postulated as oxidative stress markers^19,20^.

Another factor that has been associated with inflammation and thus with OS, confirmed in various diseases, is red blood cell (RBC) anisocytosis^21–23^, which may be expressed as a standard deviation (SD) or as a coefficient of variation (CV) of red cell distribution width (RDW)^24^. Uneven RBC size may be caused by oxidative stress, which is present after aSAH^25^. Disturbances in RBC size affect the microcirculation. Several studies have pointed to the role of the presence of microclots in cerebral infarction, and microthromboembolism has been postulated to be one of the mechanisms of DCI^26,27^. The RDW can be assessed as part of a routine complete blood count and is therefore easy to integrate into the daily routine of clinical practice. In an analysis conducted in 2022, we showed that SAH patients with poor outcomes had significantly greater RDW-CV on admission (13.9% vs 12.8%, *p* = 0.016)^28^.

Therefore, in this study, we investigated the potential value of cerebrospinal fluid (CSF) concentration of F2-isoprostanes (reflecting the oxidative stress) and erythrocyte anisocytosis (resulting in their reduced deformability, which affects the microcirculation) as predictors of DCI in aSAH patients.

## Material and methods

### Ethical statement

The local Ethical Committee of Medical University of Lodz gave a positive opinion on the research protocol (number RNN/280/13/KE). Written informed consent was obtained from patients prior to their inclusion in the study. In the case of unconscious patients, the consent was obtained in accordance with the regulations governing clinical studies as mandated by Polish law. The study was performed in accordance with Good Clinical Practice and the Declaration of Helsinki.

### Patients

**Figure 1:**
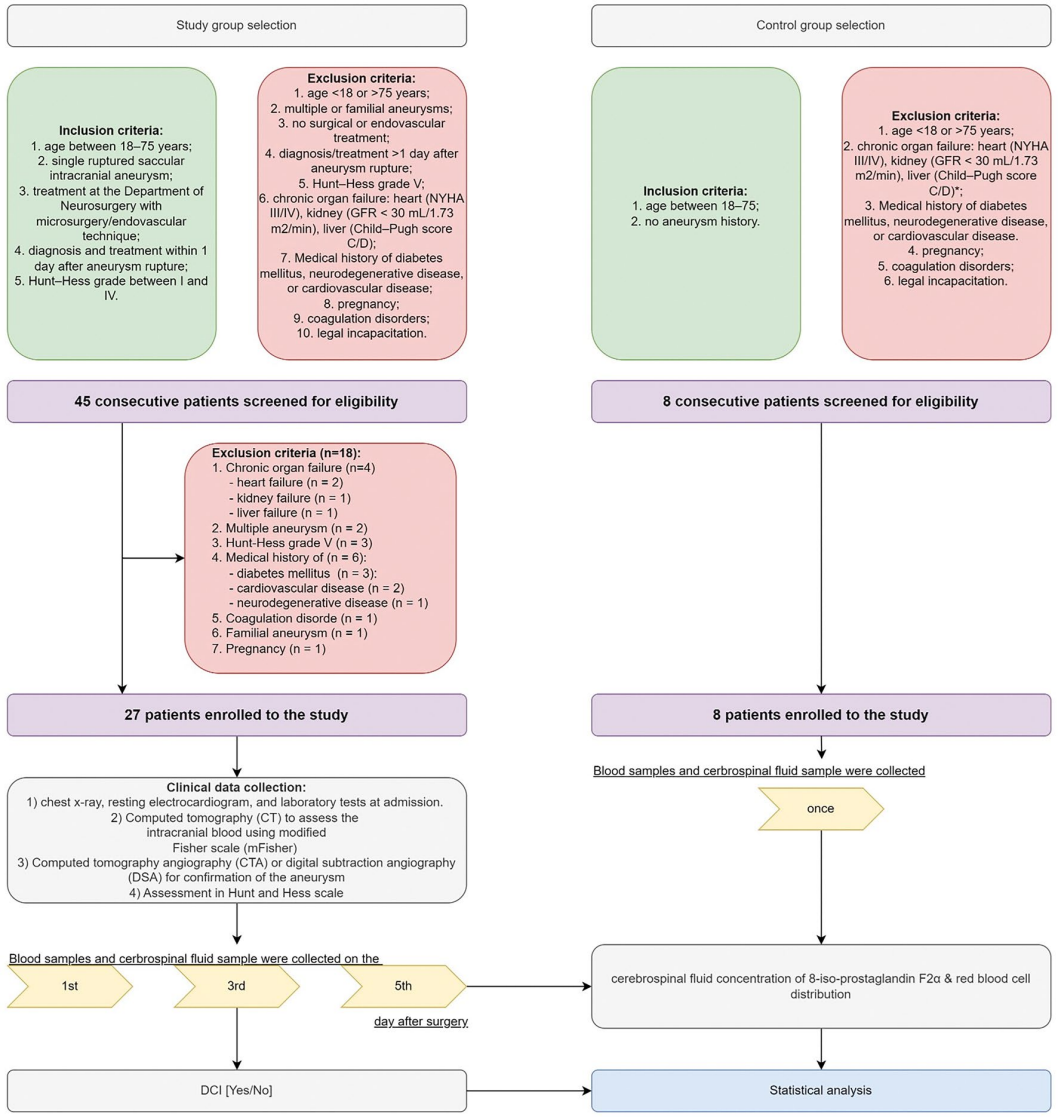
Algorithm of the study. *CT* computed tomography, *CTA* computed tomography angiography, *DCI* delayed cerebral ischemia, *DSA* digital subtraction angiography, *GFR* glomerular filtration rate, *NYHA* New York heart association classification.

We performed a prospective analysis of 27 consecutive aSAH patients. The patients were operated on for a ruptured cerebral aneurysm within 24 h after the bleeding between May 2013 and January 2018. The patient information was collected as a part of an ongoing prospective database of clinical, biochemical, and radiological data from patients with confirmed aSAH. Moreover, we included a control group consisting of 8 volunteers (4 (50%) females). The inclusion and exclusion criteria for both groups are collected in Fig. 1.

### Clinical assessment

After patient inclusion routine radiological (chest x-ray, resting electrocardiogram) and laboratory test were performed. In each case the amount of subarachnoid blood was confirmed on computed tomography (CT) according to Modified Fisher scale (mFisher) scale. Computed tomography angiography (CTA) or digital subtraction angiography (DSA) were performed to confirm the presence of the aneurysm. The patients’ clinical state was assessed according to the Hunt and Hess scale. A decision about the treatment strategy (microsurgery and endovascular intervention) was made by a multidisciplinary team. In each case we performed, routine neuroimaging examination at 12 h after aneurysm surgery.

Patients after aSAH have been subjected to the standard procedure for preventing DCI and vasospasm according to the applicable AHA recommendations^29^. Thus, in all patients after aSAH nimodypine was administered and euvolemia was maintained to prevent DCI. When clinical deterioration was observed the suspicion of DCI was taken into account. DCI was diagnosed in every case of otherwise unexplainable new symptoms of confusion or drop in the level of consciousness (by ≥ 2 point in GCS), with or without accompanying focal neurologic deficits. Therefore, the first step was to rule out other causes of patient's deterioration and to perform radiological diagnostics for the signs of vasospasm, via transcranial Doppler ultrasound and, if necessary, digital subtraction angiography. In most severe cases, in unconscious patients, intracranial pressure and cerebral perfusion pressure monitoring were used first.

The neurological examination was performed at discharge and after 1 and 12 months (in a routine out-patient clinic follow-up), by a neurosurgeon who did not have access to the study data. Glasgow Outcome Scale (GOS) and the modified Rankin Scale (mRS) were used^30,31^.

### Specimen collection

CSF was obtained via lumbar puncture on days 1, 3, and 5 post-surgery. Blood samples were drawn each time from a new venipuncture, at intervals matching the CSF sampling. The samples were aliquoted then centrifuged for 10 min at 7000*g* to remove particulates and snap frozen in liquid nitrogen, and stored at − 80 °C until further analysis. Blood samples were collected on the 1st, 3rd and 5th day after surgery, and directly passed along to the laboratory for analysis. Regarding the control group, single CSF samples and blood samples were collected and processed in exactly the same way.

### Detection of free form of F2-IsoPs (8-iso prostaglandin F_2α_)

We performed the analysis of 27 samples in the study group and in 8 samples in the control group. Free form of F2-IsoPs in CSF was quantified using STAT-8- Isoprostane ELISA Kit (Cayman Chemical, Ann Arbor, Michigan, USA). The analysis was conducted according to the manufacturer’s protocol. All samples were measured in duplicates. Synergy 2 Multi-Mode Reader (BioTek Instruments, Inc., Winooski, VT, USA) and the dedicated software were used for plate readings.

### Detection of erythrocytes anisocytosis (RDW-CV; RDW-SD)

RDW-CV and RDW-SD were analysed in routine morphology tests performed after the operation. Plasma samples (5–10 mL of morning sample taken following ≥ 8 h of fasting) were collected from each patient into commercially available ethylenediaminetetraacetic acid (EDTA) treated tubes as a part of a routine examination.

### Analytical recovery studies for 8-iso prostaglandin F_2α_, RDW-CV and RDW-SD

Analytical recovery studies were carried out using CSF samples from 3 different time points assessing the F2-IsoPs level from 3 different time points assessing the RDW-CV and RDW-SD, revealing that the recovery rate ranged from 95.9 to 97.8%.

### Statistical analysis

The analysis was performed in R statistical software (version 4.3.1)^32^ and plots were generated using the ggplot2 package^33^. Shapiro–Wilk test was used to evaluate the normal distribution, while Levene’s test was used to verify the homogeneity of variance between groups. Continuous variables with normal distribution were expressed as means ± SD and were analysed using parametric tests (ANOVA with Tukey’s post-hoc test); otherwise, as the values were expressed as medians with interquartile range and were analysed with nonparametric tests (Kruskal–Wallis test with Dunn’s post-hoc test).

The predictive potential of single factors and more complex models were assessed using receiver operating characteristic (ROC) curves with calculation of the area under the curve (AUC) (using the pROC package^34^). The optimal thresholds were identified according to Youden’s index. Finally, we performed multivariable analysis using stepwise logistic regression; the final model was evaluated for stability using the bootstrap approach (using the Generalized Linear Models and boot package^35–37^). For all analyses, the *p* values < 0.05 were considered as statistically significant.

#### Sample size calculation

The minimal sample size required for this study was determined using initial data from a cohort of the first 5 aSAH patients, who developed DCI and first 5 who did not. This calculation was based on RDW-CV, RDW-SD, and CSF ISOP measured on the first day post-surgery. We have made additional assumptions: alpha of 0.05, power of 0.8, the ratio of DCI to nonDCI patients of 2:3. Furthermore, we decided to incorporate a 10% excess into our sample size calculation to account for potential data loss or variability. Consequently n = 25 was established as the minimum number required to ensure statistical robustness and validity of the study outcomes (Table S1).

## Results

### The patients’ clinical condition

As it was shown on Fig. 1, 45 patients were screened for eligibility and 27 were included in further investigations. Their clinical and laboratory data are summarized in Table S2.

The study group comprised 16 (59.26%) males, while the control group had 4 (50%) males. The aneurysms were located most often in the anterior part of the circle of Willis (n = 24 (88.9%). DCI was diagnosed in 13 (48.15%) patients and the median day of DCI onset was day 5. The DCI diagnosis was made by a multidisciplinary team, consisting of neurosurgeons and a radiologist.

**Figure 2:**
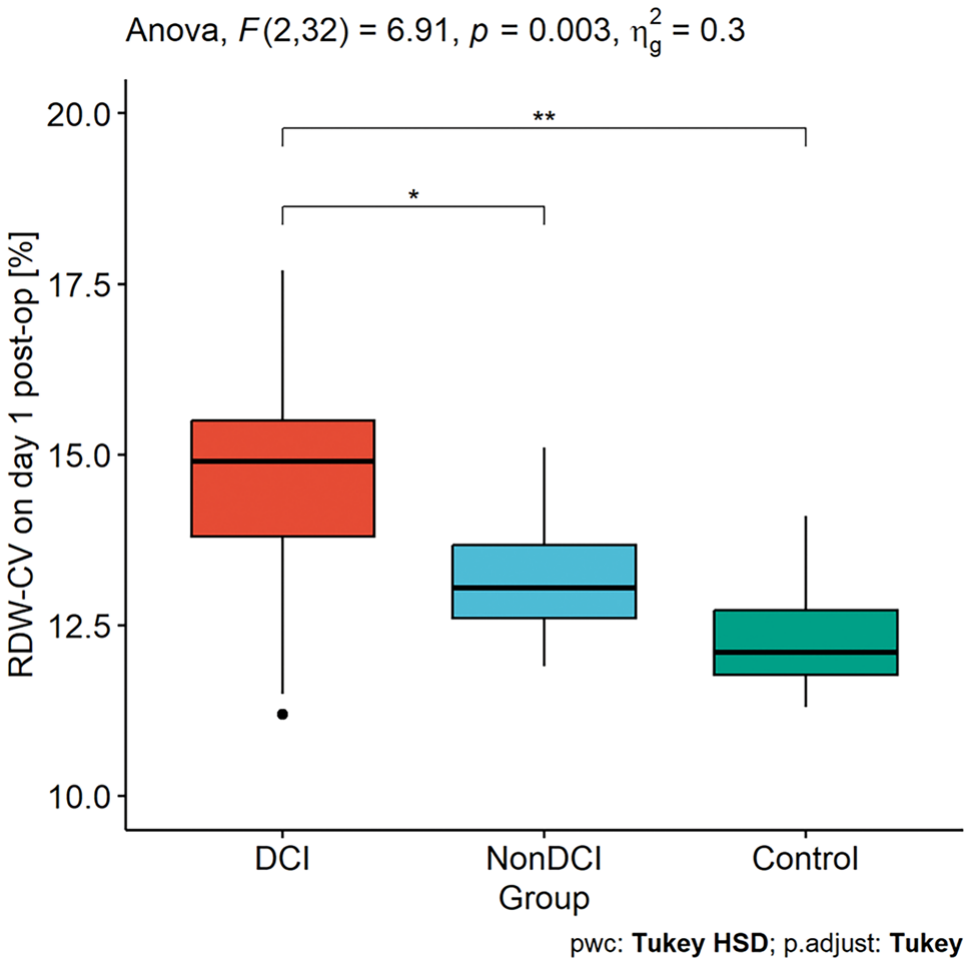
Differences in RDW-CV between the DCI, nonDCI and control groups. *DCI* delayed cerebral ischemia, *RDW-CV* red cell distribution width—coefficient of variation, *p < 0.05; **p < 0.01.

**Figure 3:**
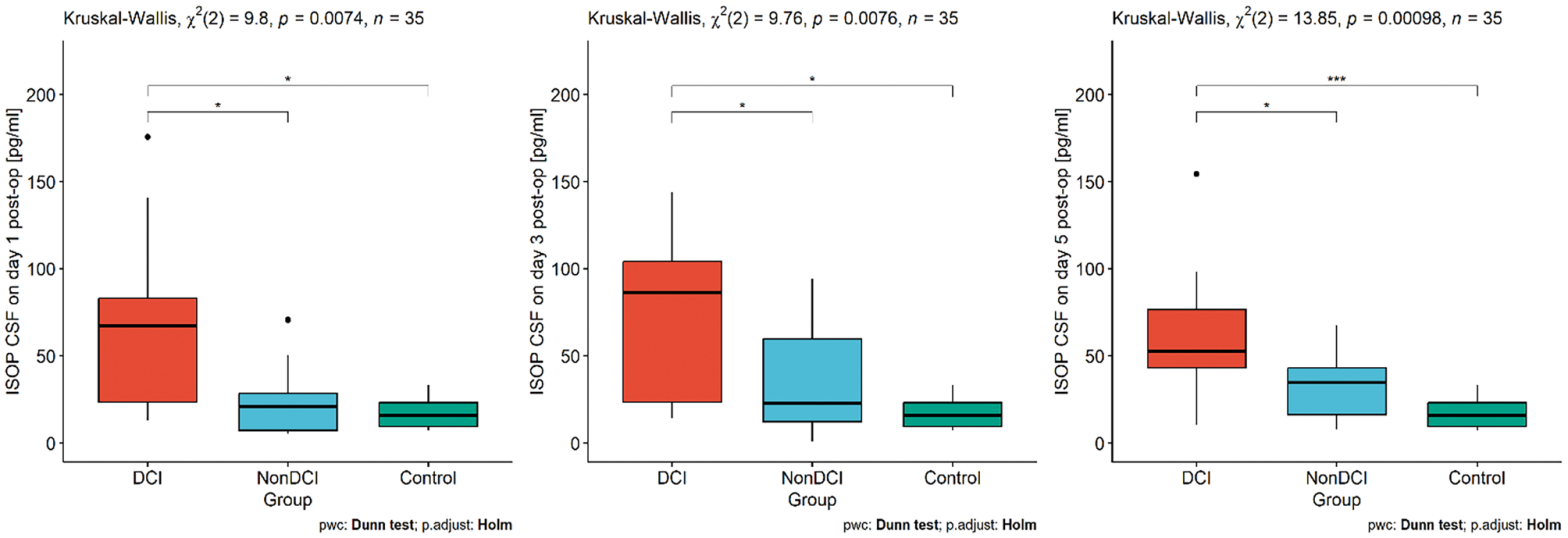
The dependencies in CSF IsoP concentration between the DCI, nonDCI and control groups measured on 1st, 3rd, 5th day after surgery. *CSF* cerebrospinal fluid, *DCI* delayed cerebral ischemia, *ISOP* 8-iso-prostaglandin F2α, *p < 0.05; **p < 0.01; ***p < 0.001.

First, we assessed the dependencies between DCI, nonDCI and the control group in terms of RDW-SD, RDW-CV and F2-IsoP levels. Analysis indicated a statistically significant difference in RDW-CV between the tested groups (see Fig. 2). Moreover, we noted that CSF F2-IsoP levels on day 1 and day 5 were associated with DCI occurrence, comparing to non-DCI patients and the controls (see Fig. 3). Interestingly, on day 3 its concentration differs only between DCI and control group (see Fig. 3).

Next, we performed a univariate analysis and assessed the relation between clinical, radiological, laboratory features and F2-IsoP levels regarding DCI occurrence. The results are presented in Table 1. In the next step, we performed a multivariate analysis (backward stepwise logistic regression). To the analysis we included only those variables that had shown to be significant in the univariate analysis. The results are presented in Table 1.

**Table 1:** The univariate and multivariate analyses assessing the relation between clinical, radiological, laboratory features and F2-IsoP levels regarding DCI occurrence.

	Total n = 27	DCI n = 13 (48.1%)	Without DCI n = 14 (51.9%)	Univariate OR (95% CI)	*P* value	Multivariate OR (95% CI)	*P* value
Age (years)	61 (50–66)	63 (61–70)	54 (43–61)	1.05 (0.99–1.12)	0.099		
Sex (male)	11 (41%)	6 (46%)	5 (36%)	1.53 (0.32–7.22)	0.582		
Location (anterior circulation)	24 (89%)	13 (100%)	11 (79%)	N.A	1.000		
Treatment (surgical treatment)	24 (89%)	13 (100%)	11 (79%)	N.A	1.000		
Hunt and Hess scale							
I	1 (4%)	0 (0%)	1 (7.1%)	1.73 (0.47–6.4)	0.405		
II	8 (30%)	3 (23%)	5 (35.8%)				
III	17 (63%)	10 (77%)	7 (50%)				
IV	1 (4%)	0 (0%)	1 (7.1%)				
mFisher grade							
I	3 (11%)	0 (0%)	3 (21.5%)	3.57 (1.14–11.13)	0.028		
II	4 (15%)	0 (0%)	4 (28.5%)				
III	4 (15%)	3 (23%)	1 (7.2%)				
IV	16 (59%)	10 (77%)	6 (42.8%)				
Intracerebral haemorrhage (ICH)	5 (18.5%)	2 (15.4%)	3 (21.4%)	0.66 (0.09–4.8)	0.687		
Hydrocephalus (HCP)	14 (51.8%)	8 (61.5%)	6 (42.8%)	2.13 (0.45–9.94)	0.334		
Anisocytosis parameters							
RDW-CV (%CV)—day 1	13.8 ± 1.5 13 (12–15)	14.5 ± 1.8 14 (13–15)	13.2 ± 0.9 13(12–13)	1.92 (1.02–3.61)	0.042	1.289 (1.041–1.597)	0.020
RDW-SD (fl)—day 1	43.5 ± 8.9 42 (39–50)	47.2 ± 10 49 (44–53)	40.1 ± 6 40 (38–42)	1.11 (1–1.23)	0.049		
RDW-CV (%CV)—day 3	12.5 ± 0.8 12 (11–13)	12.6 ± 0.9 12 (11–13)	12.4 ± 0.8 12 (11–12)	1.32 (0.55–3.16)	0.525		
RDW-SD (fl)—day 3	42.1 ± 4.5 42 (39–44)	41.6 ± 6 40 (38–44)	42.5 ± 2.5 43(40–44)	0.95 (0.8–1.13)	0.581		
RDW-CV (%CV)—day 5	12.9 ± 0.9 12 (12–13)	12.8 ± 1 12 (11–13)	12.9 ± 0.8 13 (12–13)	0.84 (0.36–1.94)	0.690		
RDW-SD (fl)—day 5	41.7 ± 4 40 (39–44)	41.5 ± 4 40 (38–43)	41.9 ± 3.6 42 (39–44)	0.97 (0.8–1.17)	0.774		
ISOP CSF conc							
Day 1 (pg/ml)	43.9 ± 42 25 (16–67)	65.7 ± 50 67 (23–83)	23.7 ± 19 20 (7–28)	1.04 (1–1.08)	0.028	1.094 (1.005–1.191)	0.039
Day 3 (pg/ml)	49.3 ± 37 43 (16–80)	65.8 ± 39 76 (21–94)	36.4 ± 30 22 (12–59)	1 (0.98–1)	0.878		
Day 5 (pg/ml)	46 ± 32 43 (22–53)	61.7 ± 52 (43–76)	31.4 ± 17 34 (16–43)	1.05 (1.004–1.097)	0.031		

**Figure 4:**
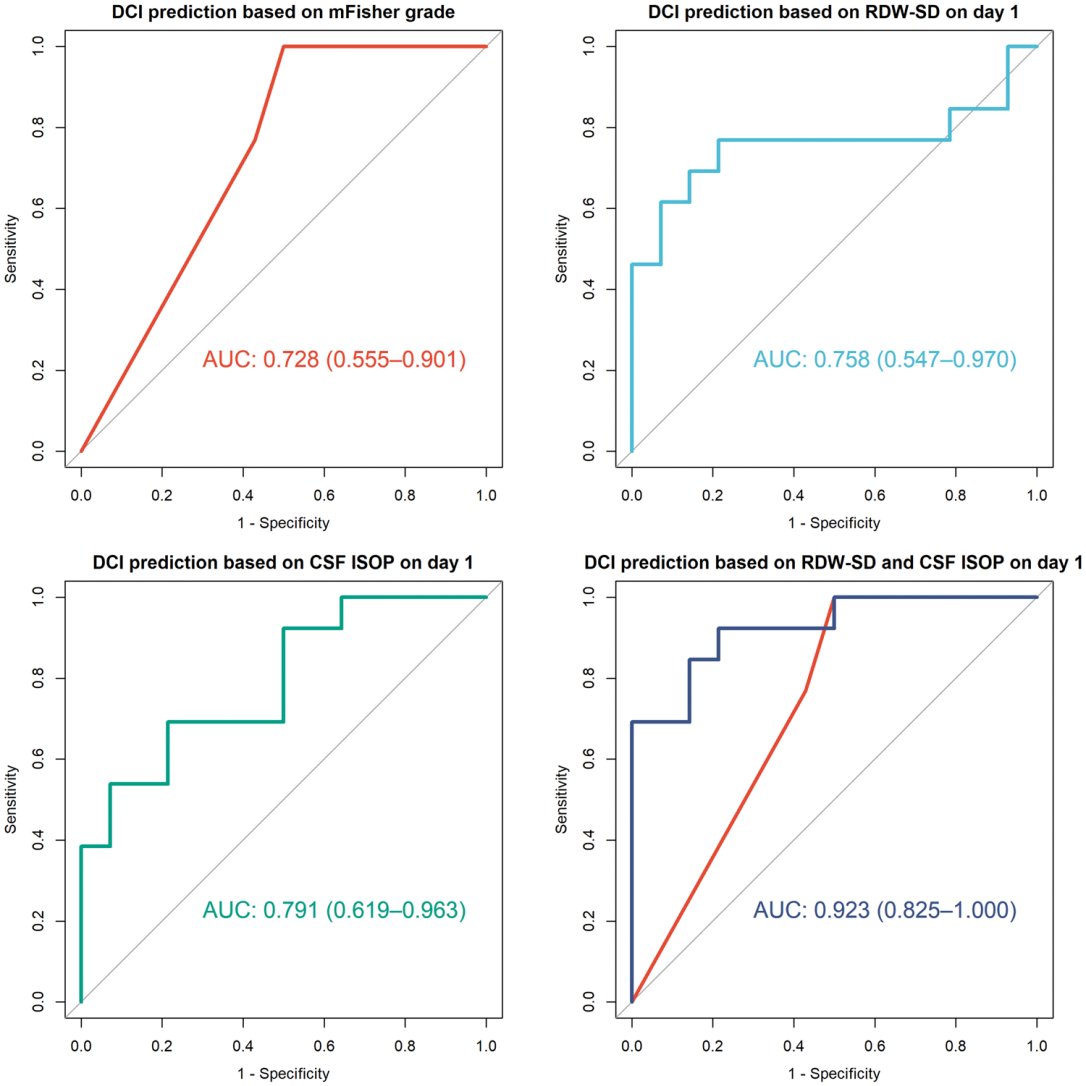
Receiver operating characteristic (ROC) curve for predicting DCI based on mFisher grade, RDW-SD on day 1 post-op and CSF F2-IsoP concentration on day 1 as well as the regression model based on both biomarkers. *AUC* area under the curve, *ISOP* F2-isoprostane.

We noted that day 1 CSF F2-IsoP concentration and day 1 RDW-SD value were the most important predictors for DCI. The receiver operating characteristic curve for DCI prediction based on multivariate models had the area under the curve (AUC: 0.923, 95% CI 0.825–1.000, *p* < 0.001; Fig. 4). To verify the stability of the model, we employed a bootstrap approach with 1000 iterations, obtaining the mean AUC value of the model of 0.919 (95% CI 0.725–0.977). In the second step we investigated the backward stepwise logistic regression models, which with a combination with the clinical features provided the highest prognostic value regarding the occurrence of DCI. ROC curves based on backward stepwise logistic regression showed that the best model for DCI prediction on the basis of the clinical markers incorporated only mFisher grade (AUC: 0.728, 95% CI 0.532–0.924, *p* = 0.023; see Fig. 4).

**Figure 5:**
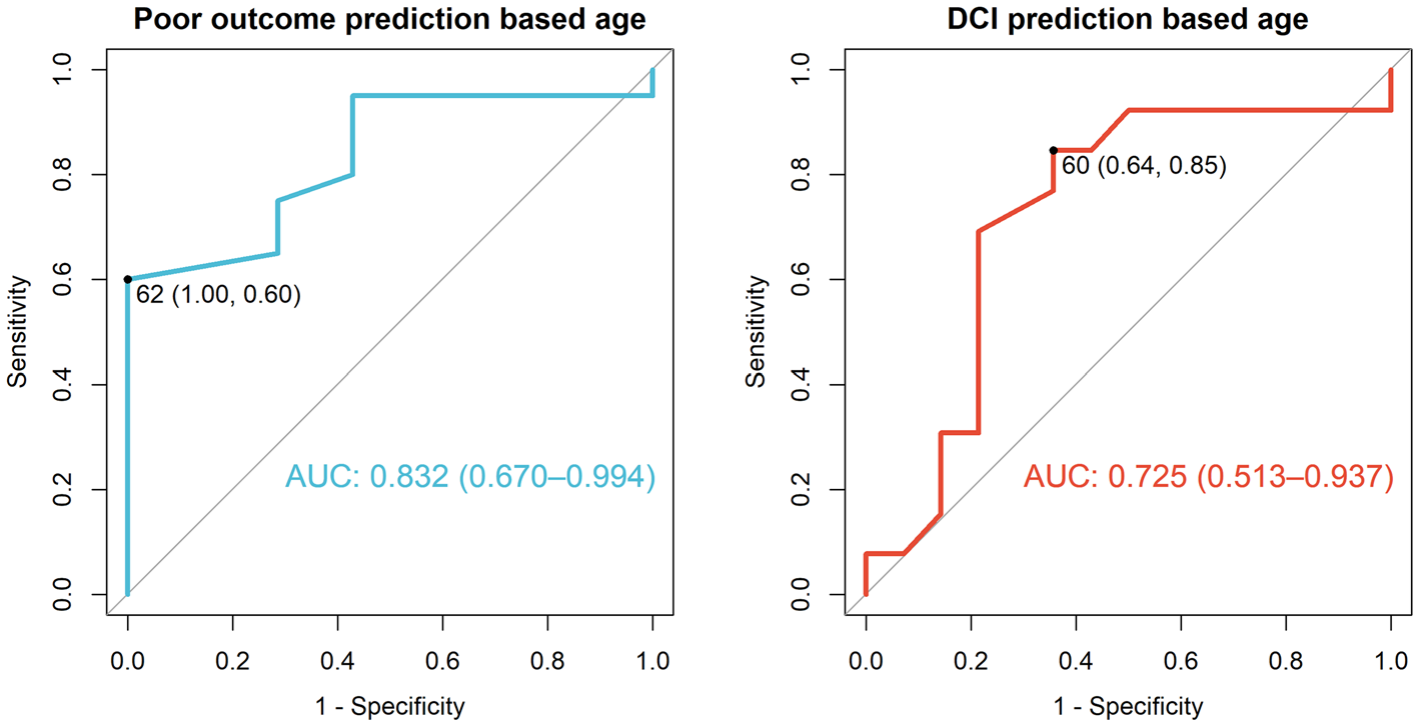
Receiver operating characteristic (ROC) curve with AUC values (with 95% confidence intervals) and optimal cut-off points (with specificity and sensitivity; based on Youden’s index) of age for prediction of: poor outcomes (in the terms of GOS at 12 month) and DCI.

Furthermore, we investigated which variables had been associated with poor long-term outcomes. To reach this purpose, we dichotomized the quality-of-life scales according to the need for assistance in everyday life (i.e., GOS scores 4–5 vs. 1–3 and mRS scores 0–3 vs. 4–6). The backward stepwise logistic regression showed that age (OR 1.1 (95% CI 1.01–1.2), *p* = 0.02) with a cut-off point of 62 years (Fig. 5), DCI (OR 23.41 (95% CI 1.8–303.7), *p* = 0.01) and male gender (OR 15.68 (95% CI 1.15–213.68), *p* = 0.03) were the most important predictors for poor outcomes according to GOS and mRS.

Moreover, we decided to investigate the correlation between the patients’ age and DCI, as according to the literature older individuals are prone to DCI^38^. According to the ROC curve and the best cut-off point was 60 years old (see Fig. 5): (AUC: 0.725, 95% CI 0.518–0.932, *p* = 0.033).

Using the same dichotomization, we also checked the correlation between the patients’ age and IsoP levels on day 5, noting a statistical trend between them. The U Mann–Whitney test result was borderline, yet non-significant (*p* = 0.07).

## Discussion

The main conclusion of this study is that elevated ISOP CSF concentration and erythrocyte anisocytosis (expressed as RDW-CV) measured on the first day after the aSAH surgery may be significant markers of an increased DCI risk. Based on this finding, we developed a predictive model for DCI occurrence incorporating both these factors. According to the literature, the model parameters meet the criteria for high predictive accuracy with AUC of 0.924 (95% CI 0.824–1.000; *p* < 0.001)^39^. Notably, we showed the potential role of oxidative stress in the DCI and predictive potential of IsoPs (as an oxidative stress biomarker) and erythrocytes anisocytosis (expressed by RDW-CV value) as an independent risk factor for DCI following aSAH.

In previous studies, it was shown that most important clinical predictors for DCI are: age (40–59 y. o.), thick SAH (high Fisher grade) and aneurysm located in the anterior circulation^40^. Our data are in line with these reports. We found that the most significant clinical parameter was the mFisher scale score (Grade 3 with 1.0 sensitivity and 0.5 specificity; Grade 4 with 0.769 sensitivity and 0.571 specificity; AUC 0.728 95% CI 0.532–0.924). However, no objective laboratory predictor is used in clinical practice. The amount of blood after SAH directly correlates with the severity of oxidative stress and DCI. It should be also mentioned that the blood in the subarachnoid space not only plays a role in the development of DCI, but also has an impact on early brain injury being the cause of neural damage in the first 72 h after the aneurysm rupture^41,42^. The extravasated blood increases intracranial pressure (ICP), leads to a decrease in the cerebral perfusion pressure (CPP) and impairs autoregulation causing cell death and brain oedema^43^. Further consequences might be microthrombosis, altered ionic homeostasis, excitotoxicity, oxidative stress and energy dysfunction secondary to cortical spreading depolarization^44–46^. All those events would add up to the DCI pathophysiology. There is no doubt that having an easily accessible biomarker of high prognostic value for DCI prediction would be of great assistance in the DCI prediction and diagnosis, on the other hand indicating a group of aSAH patients without the risk of DCI and thus reducing the cost of their hospitalization. As it seems, providing our observations are confirmed in the further studies, we could hope that our model will be useful as a biomarker for an early DCI detection.

RDW indicates that red blood cells of different sizes are present in the blood smear. RBC heterogeneity reflects the state of erythropoiesis and has been shown to correlate with inflammatory and thrombotic diseases such as ischaemic stroke^47–49^. When erythropoiesis is disturbed, RBC deformability is reduced, which impairs microcirculation^50^. Several studies have shown that RDW correlates well with in-hospital mortality, has a good ability to predict cerebral infarction and can also be used as a prognostic marker for aSAH patients^51–53^, which is in line with our findings. Subarachnoid haemorrhage causes high OS and generation of reactive oxygen species, which are directly reflected by isoprostanes formation^18,54^. The regulation of OS is particularly important for erythropoiesis^55^ with any impairment leading to elevated RDW values and microcirculatory disturbances. We therefore draw attention to OS as a cause of inflammation, microcirculatory disturbances and cerebral vasospasm after aSAH, which are all observed in DCI. We also point out that RDW and CSF isoprostanes concentration may improve the accuracy of DCI prediction with an AUC of 0.923.

At this stage of knowledge, it is difficult to point directly to the role of F2-IsoP and RDW after aSAH. We believe that both may be related to direct brain injury after aSAH leading to DCI. On the other hand, it may also show that there is a dynamic imbalance between ROS production and scavenging, leading to accumulation of OS, which may be a knowledge gap in the pathophysiology of DCI and requires future research.

The increase in oxidative stress is mostly a result of the lysis of red blood cells (RBCs) released into the subarachnoid space. Physiologically, the extravasated erythrocytes derived haemoglobin (Hb) and free heme are bound to haptoglobin (Hp) that neutralizes the strong oxidative capacity of heme–iron and prepares it for phagocytosis. In aSAH those mechanisms become rapidly exhausted and only some of the Hb and free heme originating from erythrocytes is neutralized by Hp. They collapse even faster in older patients due to insufficient antioxidant mechanisms in this age group^56^. Thus, free heme and iron in the extracellular space are the principal source of oxidative stress and reactive oxygen species (ROS).

The isoprostanes are prostaglandin-like compounds formed in vivo from the free radical-catalysed peroxidation of cell membrane arachidonic acid without the direct action of cyclooxygenase (COX) enzymes. Their concentration directly correlates with ROS production; thus, they were classified as oxidative stress biomarkers with potent biological activity^57^. In our research, we found that CSF IsoPs concentration on day 1 after surgery was elevated in the DCI group. From one side the level of IsoPs informs us about the severity of neuronal damage caused by oxidative stress as the concentration was higher in the DCI group versus non-DCI and control and from the other, their biological potential to affect the DNA synthesis in the smooth muscle cells, as well as mitogenesis and stimulation of platelets aggregation, fibroblast proliferation and vasoconstriction, all of which may also play a role in DCI pathophysiology^58^. In our work, we also showed that poor outcome and DCI occurrence were more frequently observed in older patients with the cut-off point at 60 years, as it already had been suggested in the literature^59^. The ROS formation increase is well-known to be age-related, whereas the age-dependent functional decline of antioxidant mechanisms in the population is also observed^60,61^. We would also draw attention to the trend between age and IsoPs level on day 5 as it suggests that on day 5, which was the median day of DCI onset in our cohort, there is an increase of ROS. In our opinion it is related to the insufficient antioxidant reaction that is observed in older patients. The dynamic balance between the production and elimination of ROS in patients with DCI might be shifted towards the ROS formation. This issue needs more investigation.

The pathophysiological mechanisms leading to DCI include microcirculatory disturbances and microthromboembolism. Some studies propose that anisocytosis may be associated with both of these mechanisms. According to the study by Pato et al.^62^, patients with RDW above 14% had significantly decreased RBC deformability. In an animal model study, lower blood flow was associated with reduced plasticity of RBC^63^. Although the exact mechanism remains unclear, one hypothesis proposes that erythrocytes with reduced deformability, may be recognized by macrophages, which secret the agents causing vasoconstriction and accumulation of platelets and leukocytes. Aarts et al.^64^ suggest that erythrocytes with reduced deformability were also responsible for increased platelet adhesion which could be a potential cause of aggravated microthromboembolism. However, association of RCB deformability and RDW is not fully understood as there is only one study confirming the existence of this relationship.

Many authors indicated that RDW-CV and RDW-SD show an upward trend with advanced age, however, the exact mechanism of how aging influences anisocytosis is not known^64–67^. This may potentially explain the association of RDW with poor outcome in various diseases. In our multivariate analysis, the combination of isoprostanes and RDW turned out to be a very good predictor of DCI (AUC: 0.924, 95% CI 0.824–0.1, *p* < 0.01). Additionally, RDW-SD values were also greater in patients over 59 years of age.

Anisocytosis has been identified as a risk factor of poor outcome in many diseases, also being associated with age^48^. In 2023r. Lukito et al.^53^ performed meta-analysis in which they found that high RDW value was significantly correlated with poor functional outcome, mortality and DCI. Importantly enough, our study findings are consistent with analyses involving other possible biomarkers and RDW. Researchers looking at factors such as neutrophil-to-lymphocyte ratio (NLR) or systemic inflammatory response index (SIRI) also found a correlation between RDW and DCI^68,69^. Interestingly, an analysis performed by Ingacio et al.^70^ demonstrated that RDW holds greater significance in predicting the DCI than NLR does.

A certain drawback of our work is that in the study protocol we employed the Hunt-Hess scale while WFNS scale is usually preferred in clinical analyses, albeit the Hunt-Hess scale is still widely used in clinical practice. We would like to draw attention to the fact that it is difficult to collect CFS samples at strict time points in aSAH patients. This may render incorporating suggested CSF biomarkers into the routine clinical practice somewhat difficult. Nonetheless, we believe that our efforts are sensible from a clinical point of view. Establishing a biochemical marker or screening test detecting DCI should facilitate minimally invasive DCI monitoring and prompt implementation of the treatment as well as reduce the costs of hospitalization. Finally, this could kindle a future search for a specific treatment directly preventing DCI.

## Conclusions

According to the results of our analysis, the combination of RDW-CV value and the concentration of IsoPs in CSF on the first day after surgery for a ruptured intracranial aneurysm, can serve as prognostic factors in DCI.

### Strengths and limitations of the study

In this study, we aimed to assess prognostic biomarkers for DCI following aSAH, specifically focusing on CSF 8-iso-Prostaglandin F2α and erythrocyte anisocytosis. A key strength of our study is its prospective design with prespecified inclusion and exclusion criteria, which allowed us to collect reliable data systematically until reaching the minimal sample size determined by prior calculations. Biomarker measurements were conducted at three distinct time points, and we have incorporated 12-month follow-up to enhance the robustness of our findings.

Despite these strengths, we acknowledge several limitations. This exploratory study was conducted at a single centre with the limited power of multivariate analysis, which affect the generalizability of the results. Thus, a confirmatory multicentre study with a larger sample size is necessary to confirm and expand upon our findings.

### Supplementary Information

Supplementary Table S1.

Supplementary Table S2.

## Data Availability

The datasets generated during and/or analysed during the current study are available from the corresponding author on reasonable request.
