# Supplementary material for: Evaluation of CSF 8-iso-prostaglandin F2α and erythrocyte anisocytosis as prognostic biomarkers for delayed cerebral ischemia after aneurysmal subarachnoid hemorrhage
Source: Sci Rep. 2024 May 17;14:11302. doi: 10.1038/s41598-024-61956-w (PMC11101481; doi:10.1038/s41598-024-61956-w)
Supplement: Supplementary file 1 — Supplementary Table S1. [file 41598_2024_61956_MOESM1_ESM.docx]

**Table S1**. Sample size calculation based on the data for the first 5 patients from both groups.

|  | RDW-CV [%] | RDW-SD [%] | ISOP CSF [pg/mL] |
| --- | --- | --- | --- |
| Mean for the nonDCI group | 13.42 | 35.08 | 36.55 |
| Mean for the DCI group | 15.20 | 48.90 | 91.40 |
| SD | 1.59 | 9.41 | 47.29 |
| Minimal sample size of the DCI group | 9 | 6 | 8 |
| Minimal total sample size | 25 | 17 | 22 |

Legend: CSF – cerebrospinal fluid, CV - coefficient of variation, DCI - delayed cerebral ischemia, ISOP – 8-iso-prostaglandin F2α, SD – standard deviation, RDW – red cell distribution width.
