# Supplementary material for: Evaluation of CSF 8-iso-prostaglandin F2α and erythrocyte anisocytosis as prognostic biomarkers for delayed cerebral ischemia after aneurysmal subarachnoid hemorrhage
Source: Sci Rep. 2024 May 17;14:11302. doi: 10.1038/s41598-024-61956-w (PMC11101481; doi:10.1038/s41598-024-61956-w)
Supplement: Supplementary file 2 — Supplementary Table S2. [file 41598_2024_61956_MOESM2_ESM.docx]

**Table S2.** Complete clinical data.

| **Patient number** | **Age (y.o.)** | **Sex** | **Location** | **H/H grade** | **mFisher grade** | **DCI** | **ICH** | **HCP** | **GOS at discharge** | **GOS after 12 months** | **mRS after 12 months** | **RDW-CV (%CV) day 1** | **RDW-SD (fl) day 1** | **RDW-CV (%CV) day 3** | **RDW-SD (fl) day 3** | **RDW-CV (%CV) day 5** | **RDW-SD (fl) day 5** | **ISOP CSF 1 (pg/ml)** | **ISOP CSF 3 (pg/ml)** | **ISOP CSF 5 (pg/ml)** |
| --- | --- | --- | --- | --- | --- | --- | --- | --- | --- | --- | --- | --- | --- | --- | --- | --- | --- | --- | --- | --- |
| **1** | 73 | F | A | 2 | 4 | Y | N | Y | 4 | 2 | 5 | 17.7 | 58.1 | 12.3 | 32.4 | 12.9 | 43.2 | 76.2 | 104.2 | 76.7 |
| **2** | 62 | M | A | 3 | 4 | Y | Y | N | 2 | 1 | 6 | 12.6 | 35.6 | 13.4 | 33.5 | 12.6 | 40.1 | 100.7 | 143.7 | 52.8 |
| **3** | 61 | F | A | 3 | 3 | N | N | N | 3 | 1 | 6 | 12.5 | 26.1 | 12.8 | 38.1 | 11.9 | 44.8 | 70.8 | 49.4 | 67.4 |
| **4** | 72 | F | A | 3 | 4 | Y | N | Y | 2 | 1 | 6 | 15.9 | 49.1 | 14.2 | 39.2 | 13.9 | 47.8 | 175.6 | 105.6 | 154.3 |
| **5** | 63 | F | A | 2 | 4 | Y | N | Y | 3 | 2 | 5 | 14.9 | 49.6 | 11.3 | 40.4 | 14.1 | 38.5 | 21.1 | 97.3 | 98.2 |
| **6** | 46 | M | A | 3 | 1 | N | Y | Y | 3 | 1 | 6 | 13.7 | 40.9 | 12.6 | 42.4 | 12.5 | 36.8 | 29.4 | 16.9 | 34.9 |
| **7** | 31 | M | A | 3 | 4 | Y | N | Y | 3 | 3 | 4 | 14.9 | 52.1 | 11.6 | 43.2 | 15 | 33.8 | 83.2 | 86.5 | 71.2 |
| **8** | 64 | F | A | 3 | 3 | Y | N | Y | 3 | 2 | 5 | 15.9 | 52.2 | 11.8 | 39.8 | 11.9 | 40.6 | 67.6 | 73.5 | 43.2 |
| **9** | 58 | F | A | 4 | 1 | N | Y | N | 2 | 3 | 3 | 14.8 | 35.2 | 13.5 | 45.7 | 12.1 | 39.7 | 25.5 | 94.3 | 44.3 |
| **10** | 52 | M | A | 3 | 1 | N | N | N | 3 | 1 | 6 | 12.5 | 39.5 | 12.4 | 43.2 | 13.5 | 35.9 | 50.4 | 19.1 | 44.8 |
| **11** | 64 | M | A | 3 | 4 | Y | Y | Y | 2 | 2 | 5 | 14.5 | 53.6 | 13.5 | 44.8 | 13.1 | 40.8 | 31.8 | 76.3 | 45.5 |
| **12** | 56 | F | P | 2 | 4 | N | N | Y | 3 | 4 | 3 | 13.6 | 33.7 | 11.2 | 44.9 | 14.1 | 45.8 | 6.6 | 7.2 | 7.7 |
| **13** | 70 | F | A | 3 | 3 | Y | N | N | 4 | 1 | 6 | 15.2 | 58.1 | 11.7 | 49.1 | 11.9 | 48.5 | 30.5 | 91.4 | 51.9 |
| **14** | 42 | M | P | 3 | 4 | N | Y | Y | 4 | 4 | 3 | 13 | 40.7 | 13 | 41.8 | 12.5 | 43.9 | 5.7 | 73.8 | 36.1 |
| **15** | 56 | F | A | 3 | 4 | Y | N | Y | 2 | 1 | 6 | 15.2 | 57.4 | 14 | 55.2 | 11.6 | 49.1 | 23.5 | 19.7 | 10.3 |
| **16** | 62 | M | A | 3 | 4 | Y | N | N | 2 | 1 | 6 | 13.8 | 44.5 | 12.5 | 38.7 | 12.8 | 42.9 | 12.8 | 15.9 | 17.9 |
| **17** | 73 | F | A | 2 | 2 | N | N | Y | 3 | 1 | 6 | 13.1 | 41.3 | 11.8 | 39.6 | 13.1 | 44.7 | 6.6 | 5.3 | 19.4 |
| **18** | 82 | F | A | 1 | 2 | N | N | N | 3 | 3 | 3 | 15.1 | 50.6 | 11.4 | 44.8 | 14.2 | 45.9 | 23.9 | 23.5 | 39.7 |
| **19** | 61 | F | P | 2 | 4 | N | N | Y | 4 | 4 | 2 | 12.9 | 42.3 | 12.1 | 43.9 | 11.3 | 47.1 | 5.6 | 80.7 | 8.0 |
| **20** | 60 | M | A | 3 | 4 | Y | N | N | 3 | 2 | 5 | 11.2 | 28.4 | 12.9 | 41.2 | 11.5 | 38.6 | 67.2 | 14.1 | 36.7 |
| **21** | 61 | F | A | 3 | 4 | Y | N | N | 3 | 4 | 2 | 11.5 | 28.8 | 13.1 | 38.5 | 12.7 | 37.1 | 140.7 | 120.6 | 89.6 |
| **22** | 35 | F | A | 3 | 4 | N | N | N | 3 | 4 | 3 | 13.1 | 48.9 | 14 | 44.9 | 13.1 | 39.5 | 8.8 | 0.8 | 12.3 |
| **23** | 68 | M | A | 3 | 4 | N | N | N | 3 | 2 | 5 | 13 | 44.6 | 12.6 | 43.9 | 14.1 | 39.4 | 15.8 | 22.1 | 15.1 |
| **24** | 90 | M | A | 2 | 3 | Y | N | Y | 4 | 1 | 6 | 15.5 | 47.3 | 11.8 | 45.1 | 12.9 | 38.7 | 23.6 | 23.4 | 54.8 |
| **25** | 48 | M | A | 3 | 4 | N | N | N | 3 | 3 | 4 | 12.1 | 39.9 | 13.2 | 38.1 | 13.1 | 40.1 | 17.7 | 43.5 | 34.6 |
| **26** | 42 | F | A | 2 | 2 | N | N | N | 4 | 5 | 0 | 11.9 | 38.5 | 11.4 | 40.3 | 12.9 | 39.4 | 25.6 | 10.7 | 24.9 |
| **27** | 38 | F | P | 2 | 2 | N | N | Y | 4 | 4 | 2 | 14 | 40 | 11.7 | 44.5 | 13.3 | 44.4 | 39.5 | 63.3 | 51.7 |

Legend: CSF – cerebrospinal fluid, DCI – delayed cerebral ischemia, F – female, ISOP - 8-iso-prostaglandin F2α, M – male, N – no, RDW-CV – red cell distribution width - coefficient of variation, Y – yes, A- anterior part of circle of circle of Willis, P-posterior part of circle of Willis.
